# Impact of calcium suppression imaging on hand and wrist fracture detection in spectral CT: a comparison with X-ray and CT

**DOI:** 10.1007/s00256-026-05156-9

**Published:** 2026-02-07

**Authors:** Thomas Marth, Franziska Adomat, Fides R. Schwartz, Tim S. Fischer, Reto Sutter, Anna L. Falkowski

**Affiliations:** 1 Clinic for Radiology and Nuclear Medicine, Cantonal Hospital Winterthur, Brauerstrasse 15, 8401 Winterthur, Switzerland; 2 Department of Radiology, Balgrist University Hospital, Forchstrasse 340, 8008 Zurich, Switzerland; 3 Faculty of Medicine, University of Zurich, Pestalozzistrasse 3, 8032 Zurich, Switzerland; 4 Swiss Center for Musculoskeletal Imaging, Balgrist Campus AG, Lengghalde 5, 8008 Zurich, Switzerland; 5 Department of Radiology, Brigham and Women’s Hospital, Francis Street 15, Boston, MA 02115 USA

**Keywords:** Spectral CT, Dual-layer CT, Calcium suppression, Virtual non-calcium, Fracture detection, Bone marrow edema, Upper extremity

## Abstract

**Objectives:**

To evaluate the impact of calcium suppression (CaSupp) imaging of spectral computed tomography (SpCT) on fracture detection in clinical routine in the acute trauma setting of the hand and wrist.

**Materials and methods:**

Retrospective inclusion of 125 patients who underwent both X-ray and SpCT examinations of the hand and wrist in an acute trauma setting. Two independent readers evaluated fracture presence on X-ray, conventional CT, and conventional CT plus CaSupp images (time interval 4 weeks, each). Bone bruise (BBr) presence was evaluated on CaSupp images. Sensitivity and specificity were calculated according to the consensus reading as the reference standard.

**Results:**

Of the 125 patients (mean age, 55 years ± 19.5 [SD]; 67 female), 120 presented with at least one fracture, for a total of 212 fractured bones. Sensitivity was increased significantly for both readers in CT + CaSupp (94.3 and 97.2%) compared to conventional CT (88.2 and 90.1%, *p* < .01) and X-ray (72.6 and 75.9%, *p* < .01). Specificity was 99.9 and 100% for CT + CaSupp, 99.8 and 99.9% for conventional CT, and 99.4 and 99.9% for X-ray. Reader 1 detected 13 additional fractures in CT + CaSupp compared to conventional CT alone, while Reader 2 detected 15 additional fractures. BBr was seen in 58% of all fractures, in 76% of multifragmentary fractures, and in 13% of avulsion fractures. Inter-reader *κ* was almost perfect for X-ray (*κ* = .85), CT and CT + CaSupp (*κ* = .95), and substantial for BBr (*κ* = .61).

**Conclusion:**

CaSupp images in combination with conventional CT images significantly improved sensitivity for acute trauma fracture detection in the hand and wrist compared to conventional CT images alone and X-ray.

**Supplementary Information:**

The online version contains supplementary material available at https://doi.org/10.1007/s00256-026-05156-9.

## Introduction

Spectral computed tomography (SpCT) is becoming a widely available technology, which holds the potential to reduce the diagnostic uncertainty in conventional single-energy computed tomography (SECT) [1–3]. In SECT, the voxel intensity is related to the magnitude of X-ray attenuation, which is not unique to a given material. Thus, different elemental compositions can be represented by the same pixel value on the CT grayscale (Hounsfield unit) [4, 5]. In contrast, SpCT allows for material decomposition using the variable X-ray absorption characteristics of specific materials at two different energies [4, 6]. While in SECT, a single X-ray beam is emitted from a single source and received by a single detector [3, 7], SpCT, in contrast, refers to using different X-ray energy spectra for imaging [5]. In current source-based dual-energy SpCT, it is mandatory to decide prospectively if spectral imaging is needed [2, 5, 8]. In contrast, dual-layer SpCT systems hold the advantage of acquiring spectral data inherently with each scan, as the spectral separation is achieved at the detector level, with the top layer preferentially absorbing photons of low energies and the bottom layer absorbing the remaining high-energy photons [4, 9–11].

As spectral images allow for discrimination of materials consisting of different atomic numbers, spectral images can be reconstructed to highlight specific materials, e.g., uric acid crystals in gout imaging [12], or by removing specific materials from the images, e.g., removal of calcium (Ca) in bone marrow imaging, to reveal pathologies otherwise being obscured, e.g., fractures [13]. The latter is known as calcium suppression (CaSupp) imaging or virtual von-calcium (VNCa) imaging [1, 14]. The virtual removal of calcium can potentially reveal bone marrow pathology in osseous regions. In particular, edematous changes in bone marrow after trauma can aid in detecting otherwise challenging-to-diagnose fractures. In the upper extremity, fractures of the carpal bones may be missed due to their complex anatomy and if untreated are at risk of posttraumatic complications [1, 15, 16].

However, studies that evaluated whether CaSupp imaging can improve fracture detection in the upper extremity are limited. Therefore, the aim of this study was to evaluate CaSupp images on SpCT in combination with conventional CT images compared to conventional CT images alone or X-ray images in fracture detection of the upper extremity. Our hypothesis was that CaSupp images can enhance fracture detection due to their ability to reveal bone marrow edema compared to conventional CT and X-ray, particularly in occult or challenging-to-diagnose fractures.

## Materials and methods

### Ethics approval

This retrospective single-center study was approved by the local institutional review board (Cantonal Ethics Committee, Zurich, Switzerland). It was performed according to the principles of the Declaration of Helsinki and national ethics standards. All patients signed a written general informed consent form allowing their health-related data to be used for research purposes.

### Patients

**Fig. 1:**
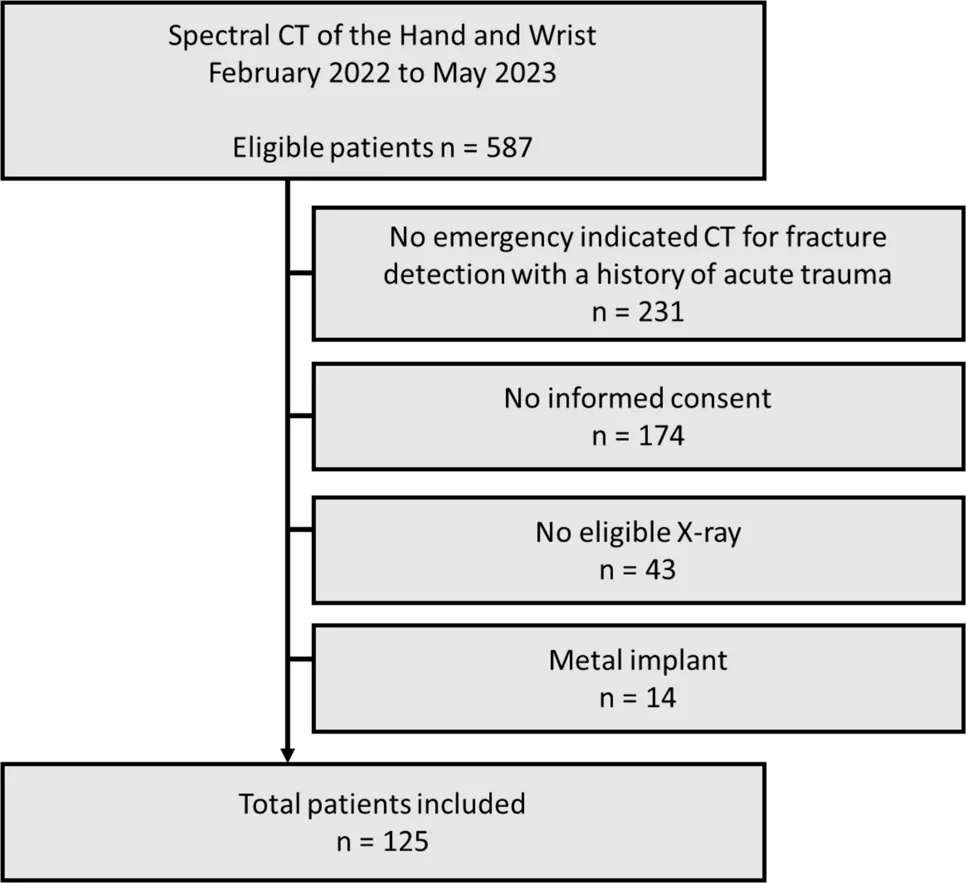
Flowchart of the inclusion process of patients with spectral CT of the hand and wrist. Of the initially 587 eligible patients, 125 were included in the study

For the retrospective identification of patients, the institutional radiology information system (RIS, medavis GmbH, Karlsruhe, Germany) was searched for patients who underwent clinically indicated CT scans of the hand or wrist on a single dual-layer SpCT scanner between February 2022 and May 2023. Inclusion criteria comprised indication for fracture diagnosis with history of acute trauma, written informed consent, and an X-ray taken within 7 days of the CT examination. Patients with metal implants in the scan area were excluded. In total, 127 SpCT examinations from 125 patients were included (Fig. 1). Patient characteristics such as age and gender were extracted from the picture archiving and communication system (Merlin, Phoenix-PACS, Freiburg im Breisgau, Germany).

### X-ray, CT, and CaSupp imaging

All images were acquired in the clinical setting and conducted using the standard clinical protocol. X-ray acquisitions were standardized in relation to the suspected fracture, e.g., dorso-volar + lateral in the wrist, dorso-volar + lateral + oblique in the carpus and metacarpus, and dorso-volar + lateral + oblique radial + oblique ulnar in suspected scaphoid fractures. All CT examinations were performed on the same SpCT scanner (CT 7500, Philips Healthcare, Best, Netherlands). The detailed CT acquisition and reconstruction parameters are listed in Table 1. CaSupp images were reconstructed from spectral-based image data using the vendor’s dedicated software (IntelliSpace Portal, Spectral Diagnostics Suite, Philips Healthcare, Best, Netherlands) with the CaSupp indices set to 76.

**Table 1:** CT acquisition and reconstruction parameters

CT acquisition and reconstruction parameters of the hand/wrist	
Scanner model	Spectral CT 7500 Philips
Tube potential (kVp)	120
Tube current–time exposure product (mAs)	79
CTDI_vol_ [mGy]	5.6
Single collimation (mm)	0.625
Total collimation (mm)	40
Pitch factor	0.2
Revolution time (s)	0.33
Exposure modulation type	none
Field of view (mm × mm)	175
Matrix	512 × 512
Axial source slice thickness (mm)	0.9
Increment	0.45
Reconstruction kernel	IMR1 SharpPlus

### Image analysis

**Fig. 2:**
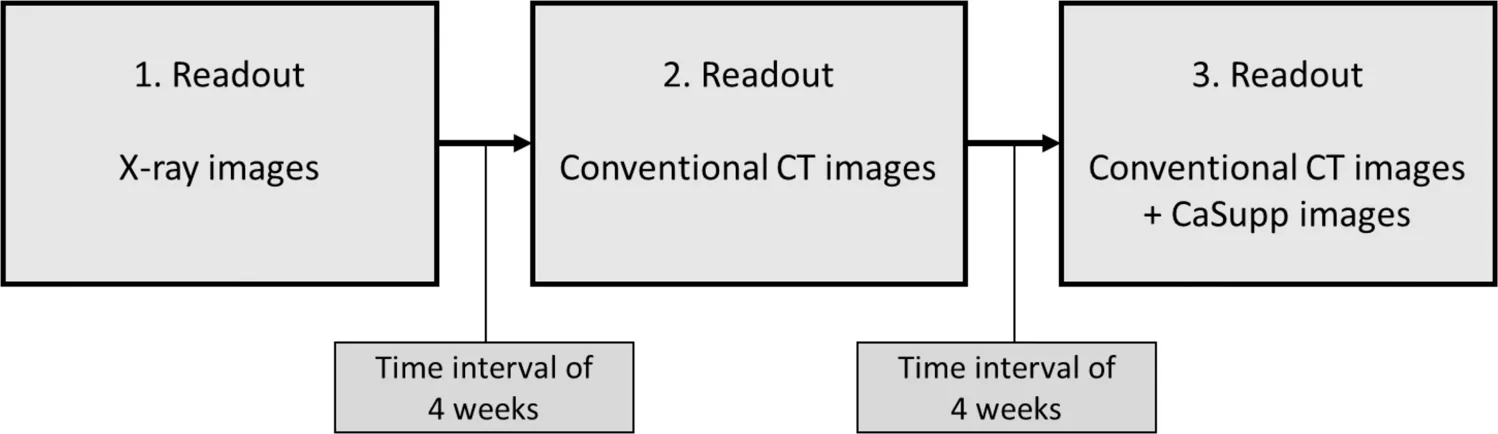
Flowchart illustrating readout scheme

Two radiologists (TM with 4 years of experience and FA with 6 years of experience) conducted three readouts on a dedicated PACS workstation with a time interval of 4 weeks between each readout. Images were anonymized and presented in random order. Readers were blinded to each other’s readout, clinical information, and imaging results. In the first readout, only X-ray images were evaluated; in the second, only conventional CT images were evaluated; and in the third, conventional CT images were evaluated in combination with CaSupp images (Fig. 2). Readers used axial, sagittal, and coronal images displayed in the bone window but were free to adjust the window level. The multiplanar reconstruction function was used by the readers in the PACS to reconstruct sagittal and coronal images from axial source images in CT and CaSupp images. Conflicting findings of fracture evaluation were resolved by consensus readout between both radiologists using X-ray, CT, and CaSupp images. The evaluation was performed similar to a different study analyzing fracture detection [17]. The following features were rated:

- fracture (yes/no),
- fractured bone (all evaluated bones are listed in Table 2),
- bone bruise (yes/no),
- multifragmentary fracture (yes/no),
- articular involvement (yes/no),
- avulsion fracture (yes/no),
- dislocated fracture (> 2 mm)
- proximal location (yes/no),
- mid location (yes/no),
- distal location (yes/no).

**Table 2:** Comparison of the fracture counts of Readers 1 and 2 and the consensus readout. The sensitivity and specificity are given for all bones for each readout in Table 3

	Reader 1			Reader 2			Consensus Readout	
Bone	X-ray	CT	CT + CaSupp	X-ray	CT	CT + CaSupp	X-ray, CT, CaSupp	% of all fractures
Radius	83	85	86	88	86	87	87	41.0%
Ulna	28	31	32	33	31	32	32	15.1%
Scaphoid	9	11	15	10	11	13	14	6.6%
Lunate	0	1	1	0	1	3	5	2.4%
Triquetrum	6	14	13	11	12	14	14	6.6%
Pisiform	0	0	0	0	0	0	0	0.0%
Trapezium	1	3	3	0	5	5	5	2.4%
Trapezoid	0	2	2	0	2	3	3	1.4%
Capitate	1	3	5	0	4	5	6	2.8%
Hamate	2	4	4	1	3	4	4	1.9%
Metacarpal I	3	3	5	5	4	5	5	2.4%
Metacarpal II	1	2	2	0	2	2	2	0.9%
Metacarpal III	1	2	2	3	2	2	2	0.9%
Metacarpal IV	5	7	7	5	7	7	7	3.3%
Metacarpal V	8	10	9	8	10	9	9	4.2%
Dig.I prox. phalanx	0	0	0	0	0	0	0	0.0%
Dig.I dist. phalanx	0	2	2	0	2	2	2	0.9%
Dig.II prox. phalanx	1	1	1	1	1	1	1	0.5%
Dig.II med. phalanx	1	1	1	1	1	1	1	0.5%
Dig.II dist. phalanx	1	1	1	1	1	1	1	0.5%
Dig.III prox. phalanx	1	1	1	1	1	1	1	0.5%
Dig.III med. phalanx	2	2	2	2	2	2	2	0.9%
Dig.III dist. phalanx	0	1	1	1	1	1	1	0.5%
Dig.IV prox. phalanx	1	1	1	1	1	1	1	0.5%
Dig.IV med. phalanx	0	0	0	1	1	1	1	0.5%
Dig.IV dist. phalanx	1	1	1	2	1	1	1	0.5%
Dig.V prox. phalanx	0	1	1	2	0	0	1	0.5%
Dig.V med. phalanx	3	3	3	3	3	3	3	1.4%
Dig.V dist. phalanx	0	1	1	1	1	1	1	0.5%
All bones	159	194	202	181	196	207	212	100.0%

*CT* computed tomography, *CaSupp*, calcium suppression

On X-ray, CT, and CaSupp images, a fracture was defined as a disruption of bone, identified by cortical/and or trabecular discontinuity. Bone bruise was only evaluated in CaSupp images and was defined as a patchy or geographic area of increased trabecular attenuation, independent of the presence of an associated fracture.

### Statistical analysis

Continuous variables were assessed for normal distribution with the Shapiro–Wilk test and homogeneity of variances with the Levene test. If distributed normally, continuous data are presented as a mean with a standard deviation (SD). Sensitivity and specificity were compared among different image sets with the McNemar test and corrected for multiple pairwise comparisons with the Bonferroni procedure. Interreader agreement was assessed using kappa coefficients and given with the 95% confidence interval (CI). Kappa (*κ*) was categorized according to the system of Landis and Koch [18]: less than 0.200, slight agreement; 0.200–0.399, fair; 0.400–0.599, moderate; 0.600–0.799, substantial; and 0.800 or greater, almost perfect. All statistical analyses were performed using SPSS Statistics (version 29, IBM) and RStudio (V4.5.1, The R Foundation).

## Results

### Patient demographics

A total of 125 patients who underwent X-ray and SpCT examinations within 7 days (mean 0.1 days, range from −6 to + 6) for fracture diagnostic of the hand or wrist following a history of acute trauma were included. The left side was examined in 71 patients, the right side in 52 patients, and both sides in two patients (both males). All patients were over 18 years of age, with a mean age of 55 years ± 19.5 (SD). The distribution between females and males was 67 (52.8%) to 60 (47.2%).

### Image analysis–fracture evaluation

On X-ray imaging, reader 1 rated a total of 159 bones as fractured, while reader 2 rated 181 bones as fractured. Sensitivity was 72.6% for reader 1 and 75.9% for reader 2, while the specificity was 99.9% for reader 1 and 99.4% for reader 2. Interreader agreement was almost perfect (*κ* = 0.85; 95% CI: 0.81–0.89). The most common fractured bone, classified by reader 1 and reader 2, was the radius (83/88), followed by the ulna (28/33), the scaphoid (9/10), the triquetrum (6/11), the metacarpal V (8/8), and the metacarpal IV (5/5).

On conventional CT images, reader 1 rated a total of 194 bones as fractured, while reader 2 rated 196 bones as fractured. Sensitivity was 88.2% for reader 1, and 90.1% for reader 2, while the specificity was 99.8% for reader 1 and 99.9% for reader 2. Interreader agreement was almost perfect (*κ* = 0.95; 95% CI: 0.93–0.97).

**Fig. 3:**
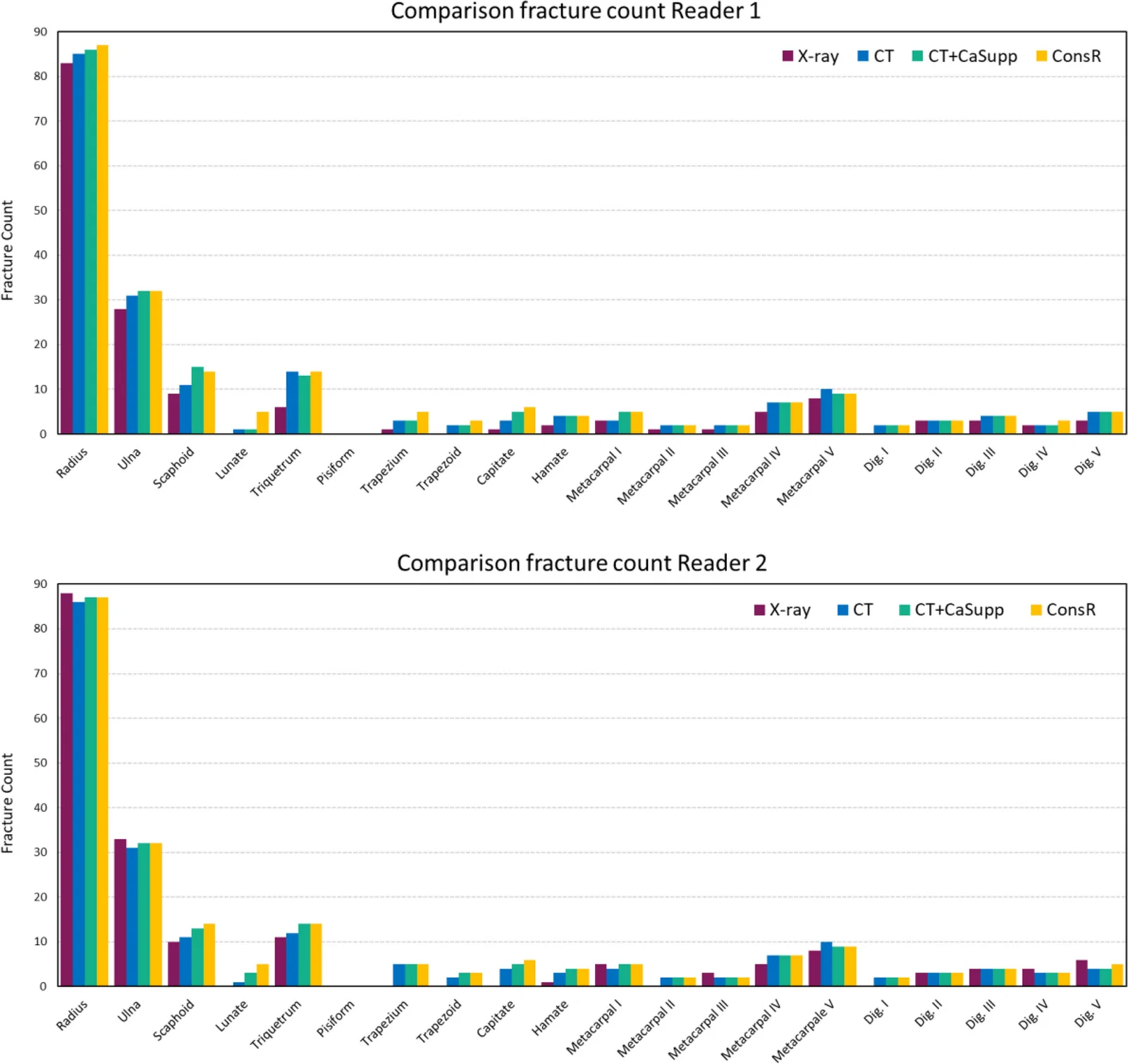
Comparison of fracture count on X-ray (red), CT (blue), CT in combination with calcium suppression (CaSupp) images (green), and in the consensus readout (ConsR; yellow)

**Fig. 4:**
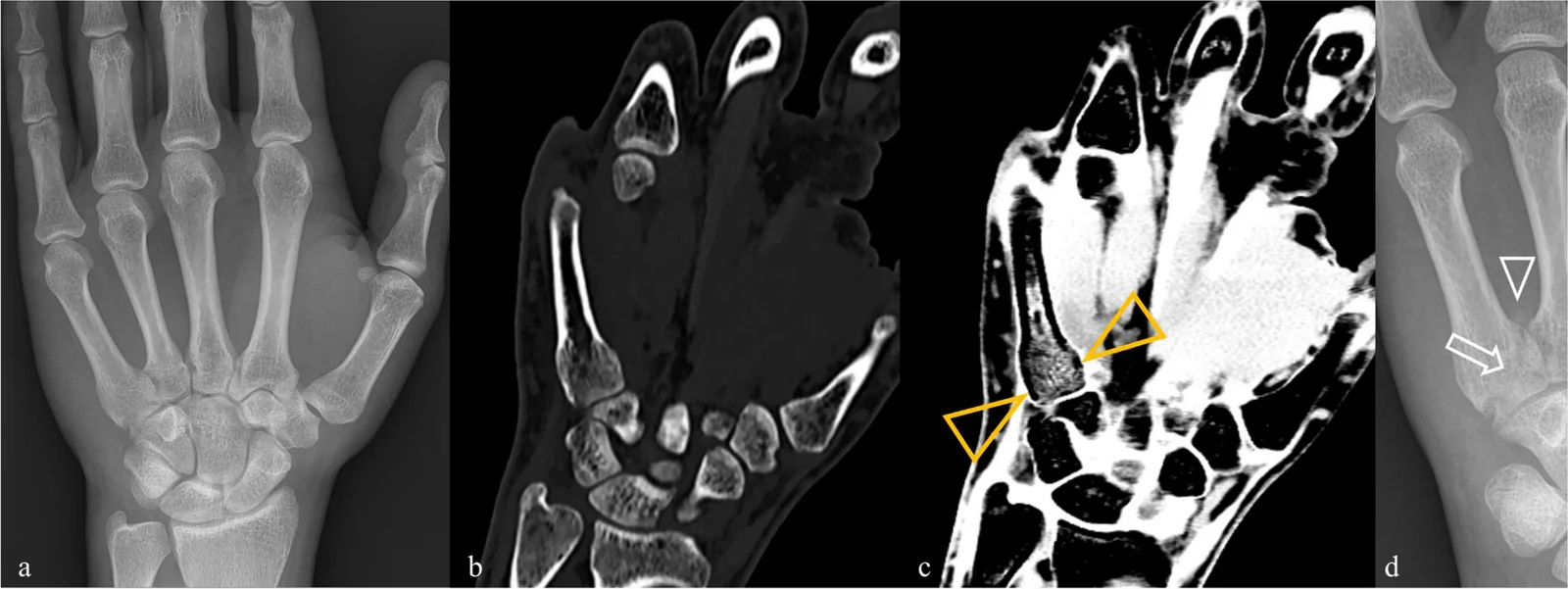
Dorsovolar X-ray (**a**, **d**) and coronal CT images (**b**, **c**) in a 26-year-old man after a fall on the left hand. The initial X-ray (**a**) and conventional CT (**b**) revealed no fracture. CaSupp images (**c**) demonstrated bone marrow edema (yellow arrowheads) at the base of the fifth metacarpal bone, prompting both readers to report a non-displaced fracture in contrast to the X-ray and conventional CT report. In the follow-up X-ray (**d**), 9 days after initial imaging (**a**–**c**), the hyperlucent fracture line (white arrow) is well depicted, accompanied by callus formation (white arrowhead)

**Fig. 5:**
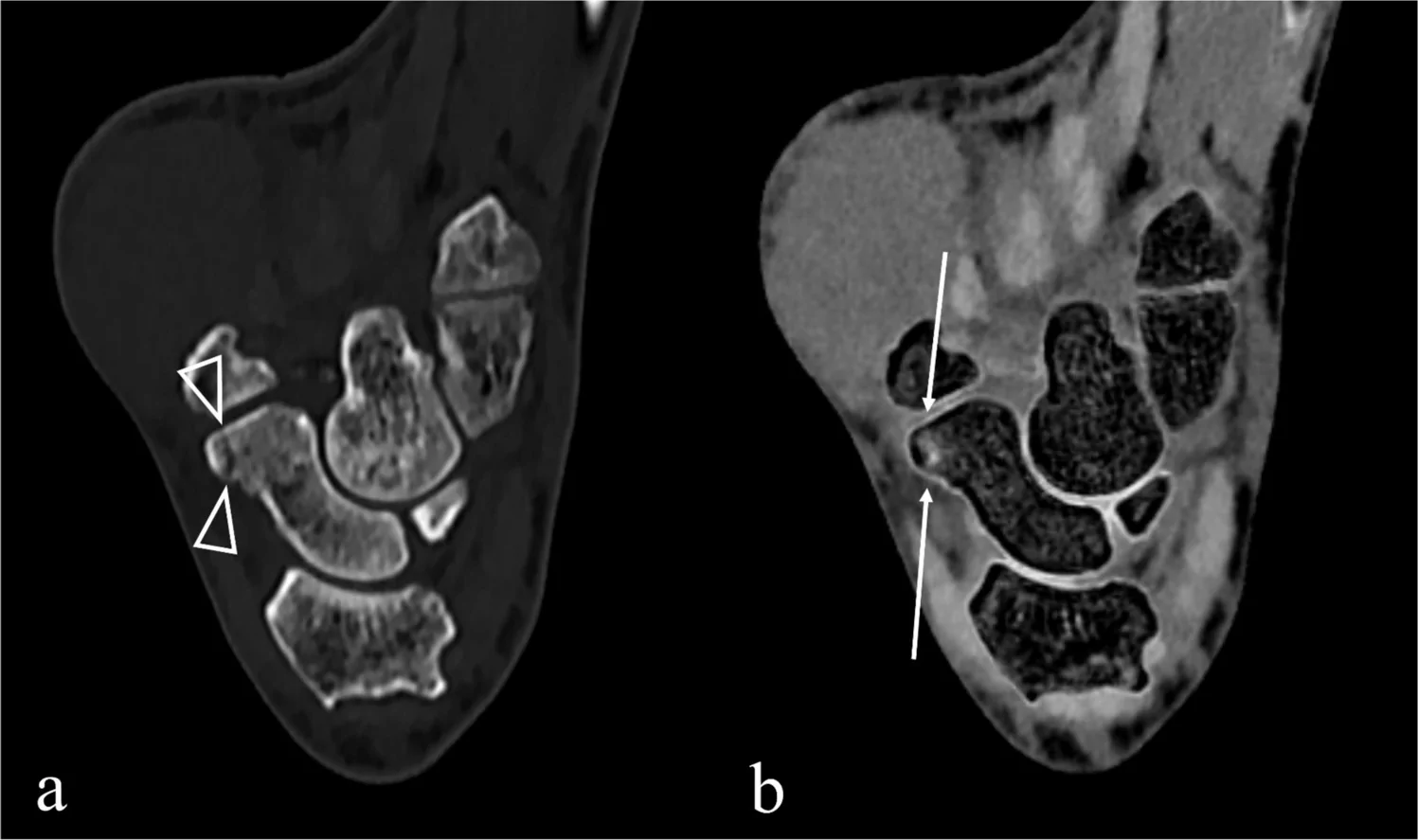
Conventional CT image (**a**) and CaSupp image (**b**) in a 24-year-old male after a fall on the right hand, shown in an oblique coronal reconstruction. While only a very light bone bruise can be seen around the small non-displaced fracture of the distal scaphoid pole (arrows) in the CaSupp image, the fracture gap was well visible, while it was missed on conventional CT imaging by both readers (arrowheads)

When using conventional CT in combination with CaSupp images, reader 1 rated a total of 202 bones as fractured, while reader 2 rated 207 bones as fractured. Sensitivity was 94.3% for reader 1, and 97.2% for reader 2, while the specificity was 99.9% for both readers. Interreader agreement was almost perfect (*κ* = 0.95; 95% CI: 0.93–0.97). A detailed list of detected fractures for each reader, readout, and bone is shown in Table 2. Sensitivity, specificity, and *p*-values for comparison between readouts are reported in Table 3. Sensitivity was significantly higher in CT + CaSupp compared to CT and X-ray images for both readers (*p* < 0.01). Specificity showed no significant difference between CT + CaSupp and CT images for both readers (*p* = 0.38). In the consensus readout, of 125 patients, 120 were diagnosed with a fracture, with a total of 212 fractures. The comparison of the results of the different readouts is illustrated in Fig. 3. CT and CaSupp images are shown in Figs. 4 and 5.

**Table 3:** Sensitivity and specificity of X-ray, CT, and CT + CaSupp readouts

	Reader 1			Reader 2		
	Fracture count	Sensitivity	Specificity	Fracture count	Sensitivity	Specificity
X-ray	159	72.6%	99.9%	181	75.9%	99.4%
CT	194	88.2%	99.8%	196	90.1%	99.9%
CT + CaSupp	202	94.3%	99.9%	207	97.2%	99.9%
*p*-value						
X-ray vs CT		<.01*	>.99		<.01*	<.01*
X-ray vs CaSupp		<.01*	>.99		<.01*	<.01*
CT vs CT + CaSupp		<.01*	0.38		<.01*	0.38

*P*-values are given for comparison of sensitivity and specificity among different image sets by the McNemar test, corrected for multiple comparisons by the Bonferroni procedure. The asterisk (*) marks significant differences between two image sets. CT computed tomography, CaSupp Calcium Suppression

### *Additional fractures found using CT* + *CaSupp compared to CT alone*

**Fig. 6:**
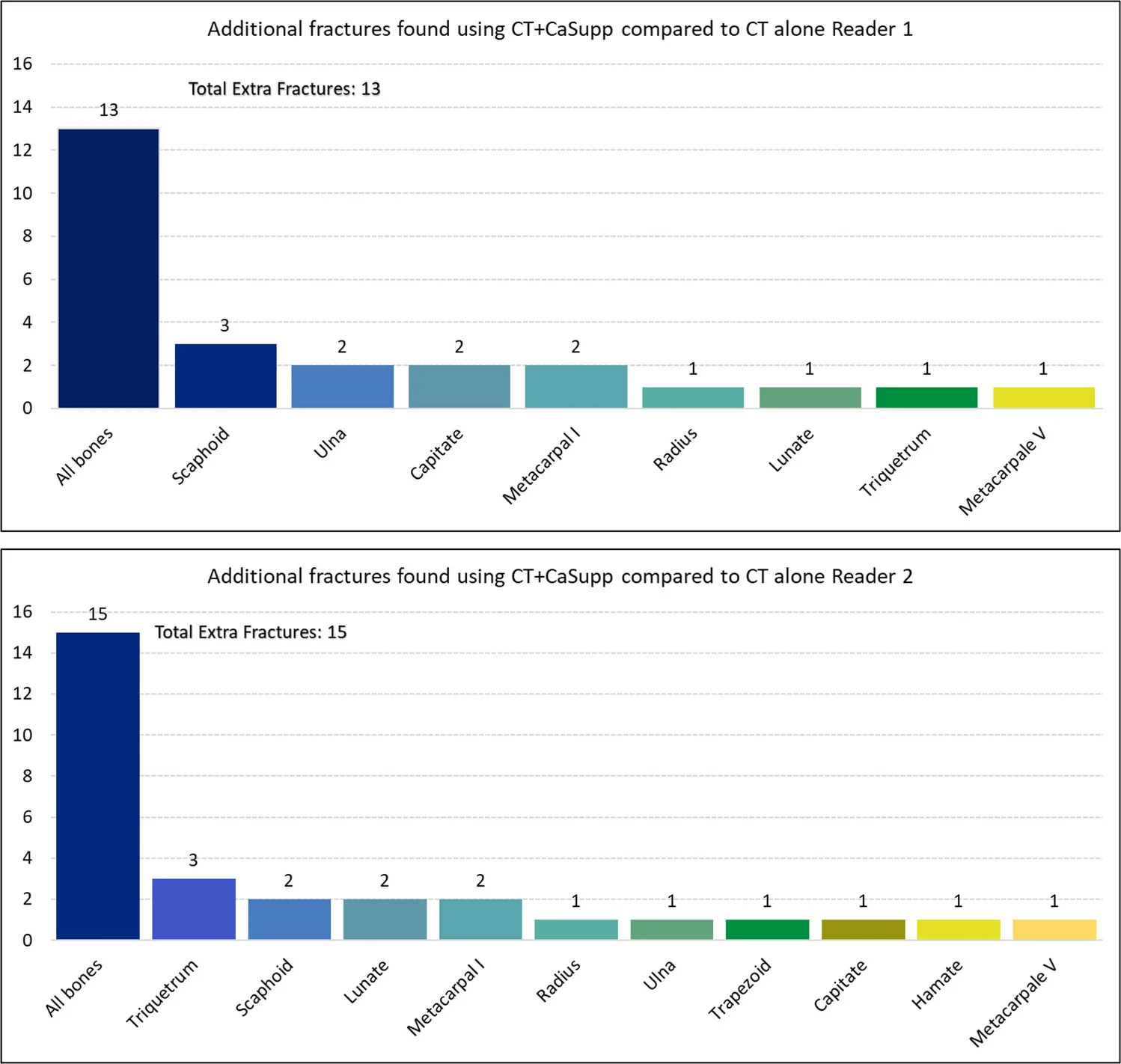
Numbers of additional fractures found in the hand and wrist using CT + CaSupp images compared to CT images alone, for reader 1 and 2, respectively

When using CaSupp images alongside conventional CT, compared to conventional CT images alone, reader 1 identified a total of 13 additional fractures (three scaphoid, two capitate, two metacarpal I, two ulna, one lunate, one triquetrum, one radius, and one metacarpal V fracture), while reader 2 found 15 additional fractures (three triquetrum, two lunate, two scaphoid, two metacarpal I, one ulna, one radius, one trapezium, one capitate, one hamate, and one metacarpal V fracture), shown in Fig. 6. The features of the additional fractures found are listed in Table 4, with the highest percentage of the additionally found fractures being articular fractures, 47–62%.

**Table 4:** Image features of additional fractures found on CaSupp plus CT images not found on conventional CT images alone

			Fracture features							
Bone		Fractures	Bone bruise	Multifragmentary fracture	Articular involvement	Avulsion fracture	Dislocated fracture	Proximal location	Mid location	Distal location
Reader 1										
	Radius	1	1	1	1	0	0	0	0	1
	Ulna	2	2	1	0	2	2	0	0	2
	Scaphoid	3	3	0	2	0	0	0	1	2
	Lunate	1	1	0	1	1	0	0	0	1
	Triquetrum	1	0	0	1	0	0	0	0	1
	Capitate	2	1	0	1	1	1	1	0	1
	Metacarpal I	2	1	1	2	0	0	2	0	0
	Metacarpal V	1	1	0	0	0	0	1	0	0
	All bones	13	77%	23%	62%	31%	23%	31%	8%	62%
Reader 2										
	Radius	1	1	0	0	0	0	0	0	1
	Ulna	1	0	1	0	1	1	0	0	1
	Scaphoid	2	2	0	1	0	0	0	1	1
	Lunate	2	0	0	2	2	0	0	0	2
	Triquetrum	3	0	0	1	2	0	1	1	1
	Trapezoid	1	0	0	0	1	0	0	0	1
	Capitate	1	0	0	0	1	1	0	0	1
	Hamate	1	0	1	1	0	1	0	0	1
	Metacarpal I	2	2	1	2	0	0	2	0	0
	Metacarpal V	1	1	0	0	0	0	1	0	0
	All bones	15	40%	20%	47%	47%	20%	27%	13%	60%

### Bone bruise and fracture features

Bone bruise was rated to be present in case of fracture in 67% by reader 1 and in 66% by reader 2. In 58% of fractures (122/212), both readers agreed that a bone bruise was present, and in 25% of fractures (53/212), both readers agreed that no bone bruise was present. In 17% (37/212), readers disagreed whether a bone bruise was present or not. Interreader agreement for bone bruise in case of fracture was substantial (*κ* = 0.61; 95% CI: 0.50–0.72). Bone bruise, as rated by both readers to be present, was seen in 76% of multifragmentary fractures (93/123), 68% of fractures with articular involvement (108/159), 64% of dislocated fractures (87/136), and in 13% of avulsion fractures (8/61). Bone bruise was present in all three evaluated fracture locations: proximal 42%, mid 60%, and distal 62%. The evaluation of bone bruise distribution in the hand and wrist fractures is listed in the Supplementary Table 1.

## Discussion

CaSupp images in combination with CT images significantly improved the sensitivity in routine diagnostics for fracture detection of the hand and wrist compared to CT images alone and X-ray images. Both readers improved their fracture detection when CaSupp images were used as part of the readout, with 13 and 15 additional fractures found, respectively, to conventional CT images alone. In particular, additional fractures of the carpal and metacarpal bones were found with CaSupp images, with 47–62% being articular fractures, 31–47% avulsion fractures, and 20–23% dislocated fractures, indicating CaSupp images to be most useful in the detection of articular carpal and metacarpal fractures.

In contrast, the specificity was not significantly improved when using CaSupp images in combination with conventional CT images compared to conventional CT images alone because the specificity of conventional CT was already very high (99.8–99.9%). The expected benefit of CaSupp images does not lie in the ability to overrule fracture diagnosis made in conventional CT, but in the detection of subtle and occult fractures otherwise missed in conventional CT, due to the highlighting effect of bright bone marrow edema surrounding fractures and fracture gaps on gray scale images.

Missed fractures in the wrist are common, as it accounts for the area with the most missed fractures, although not in relation to their absolute occurrence, but due to the absolute high number of wrist fractures compared to fractures of other anatomical regions [19]. The most challenging fractures in the wrist include fractures of the carpal bones, but missed fractures of the distal radius or the metacarpals have also been reported [20, 21]. Karl et al. showed that advanced imaging in suspected scaphoid fractures in the setting of negative radiographs represented a cost-effective strategy to reduce morbidity. With the inherently available spectral CT data images in dual-layer detector systems, CaSupp images may further improve cost-effectiveness [15, 22]. While Koch et al. and Xie et al. demonstrated that bone marrow edema can be seen in scaphoid fractures with SpCT, data on improvement in fracture detection rate are limited [22]. In our study, bone bruise was rated to be present in the case of a fracture by reader 1 in 67%, and by reader 2 in 66%, while in the subset of additional fractures found in CT + CaSupp compared to CT images alone, bone bruise was rated to be present in 77% and 40% by reader 1 and 2, respectively. While the 77% of reader 1 indicate the potential benefit of bone bruise for fracture detection, the relatively low bone bruise rate of 40% noted by reader 2 does not equally support this hypothesis, pointing at additional reasons. If we look at CaSupp images, the benefit in fracture detection may not lie only in detecting fractures by bone bruise as seen inside the bone, but CaSupp images might also highlight edema in the fracture gap. Kim et al. evaluated the diagnostic utility of spectral dual-layer CT in wrist injuries, comparing it with MRI [23]. In line with our results (sensitivity and specificity 94.3–97.2%/99.9–100% of CT + CaSupp, compared to 88.2–90.1%/99.8–99.9% conventional CT), they stated that SpCT with VNCa reconstruction is a promising tool for identifying occult wrist injuries and non-fracture contusion injuries in patients with wrist traumas. In particular, they found that an increased sensitivity for diagnosis of fractures in the radius and carpal bones (sensitivity and specificity for detecting radius fractures were 98.1%/93.8% for dual-layer CT and 96.3%/93.8% for standard CT, and for carpal bone fractures 100%/98.9% for dual-layer CT and 93.8%/100% for standard CT) could be achieved with VNCa images compared to standard CT images alone. The systematic review and meta-analysis from Bäcker et al. revealed that 21.8% of scaphoid fractures were missed on X-ray but could be diagnosed on advanced imaging: the highest sensitivities and specificities for diagnosing scaphoid fractures were reported for MRI at 94.2% and 97.7%, respectively, followed by CT with a sensitivity and specificity of 81.5% and 96.0% [24]. Our institution routinely performs all CT examinations with SpCT since February 2022. Because the detector-based spectral CT scanner always generates SpCT datasets, the images can be re-evaluated retrospectively at any time with different software tools for specific material characterizations. Therefore, even if specific clinical questions arise regarding spectral imaging after the exam, the exam does not need to be repeated; instead, it simply requires re-evaluation with different software algorithms or ideally simultaneous reconstruction of CaSupp images. Thus, the patient’s radiation dose can be reduced, and therapeutic approaches can be re-adapted before complications occur.

Additionally, undetected and thus untreated fractures may lead to delayed healing/nonunion, chronic pain or disability, and augmented financial costs due to repeated imaging, follow-up appointments, and loss of workforce. Thus, additional fractures revealed by CaSupp images might have a direct socioeconomic impact [25].

Although SpCT CaSupp images can be acquired without an increase in radiation dose, the additional image interpretation time must be considered. While not formally measured, the experience of the two readers suggests that image evaluation may require an additional 1 to 2 min per examination.

While the substantial interreader agreement observed for bone bruise in the presence of fracture (*κ* = 0.61) might be attributed to a learning effect. Another possible explanation is that it likely reflects the relatively subjective nature of bone bruise definition as an area with altered trabecular attenuation, which inherently permits a degree of interpretative variability.

Our study has some limitations: First, the analyzed fractures showed a wide range of variability. Thus, a comparison with clinical outcome was not feasible. However, our goal was to investigate the overall diagnostic value of fracture detection using CaSupp in SpCT technique. Second, we only evaluated acute fractures. Thus, we cannot report the accuracy of fracture healing analysis. Third, these results only reflect the performance of CaSupp analyses for distal upper extremity fractures, while the diagnostic performance in other skeletal regions still needs to be evaluated. Additionally, as we aimed to assess CaSupp images in routine clinical fracture diagnosis, almost no patients had received additional MRI scans. Therefore, a comparison with MRI was not feasible, particularly with respect to determining whether MRI would have identified additional fractures beyond those detected on CT with CaSupp images. Furthermore, it remains unclear whether, in cases of negative radiography and CT findings, additional evaluation with dual-layer CaSupp images or MRI should be performed. In regard to the reference standard, surgical or histopathological correlation, considered the gold standard for most diagnostic accuracy studies, was not applicable in this setting, due to the high number of conservatively treated fractures. Although commonly used in diagnostic accuracy studies, the use of a consensus reading as the reference standard may introduce consensus and incorporation bias, potentially leading to an overestimation of diagnostic accuracy.

## Conclusion

CaSupp images, when combined with conventional CT images, significantly improved the sensitivity of fracture detection in the hand and wrist in the setting of acute trauma and revealed additional fractures missed on X-ray and conventional CT images alone. In particular, fractures of the carpal and metacarpal bones could be revealed due to the presence of bone marrow edema in CaSupp images. As missed fractures may lead to poor patient outcomes and high costs for healthcare systems, the reconstruction and evaluation of CaSupp images from the inherently available spectral raw data in spectral dual-layer CT systems are recommended for fracture diagnosis in clinical routine, holding the potential for improved patient diagnosis, treatment, and socioeconomic benefits.

## Supplementary Information

Below is the link to the electronic supplementary material.

ESM 1 (DOCX 33.7 KB)

## Data Availability

Upon request, authors can send documentation or raw data in order to verify the validity of their results.

## Abbreviations

BBr: Bone bruise
Ca: Calcium
CaSupp: Calcium suppression
CT: Computed tomography
MRI: Magnetic resonance imaging
PACS: Picture archiving and communication system
SECT: Single-energy computed tomography
SpCT: Spectral computed tomography
VNCa: Virtual non-calcium
