# Supplementary material for: Impact of calcium suppression imaging on hand and wrist fracture detection in spectral CT: a comparison with X-ray and CT
Source: Skeletal Radiol. 2026 Feb 7;55(6):1297–307. doi: 10.1007/s00256-026-05156-9 (PMC13649893; doi:10.1007/s00256-026-05156-9)
Supplement: Supplementary file 1 [file 256_2026_5156_MOESM1_ESM.docx]

|  |  |  |  |  |  |  |  | Multifragmentary fracture | | | Articular involvement | | | Avulsion fracture | | | Dislocated fracture | | | Proximal location | | | Mid Location | | | Distal location | | |
| --- | --- | --- | --- | --- | --- | --- | --- | --- | --- | --- | --- | --- | --- | --- | --- | --- | --- | --- | --- | --- | --- | --- | --- | --- | --- | --- | --- | --- |
| Bone | Fractures | BBr R1 | % | BBr R2 | % | BBr_R1-R2_ | % | Total | BBr_R1-R2_ | % | Total | BBr | % | Total | BBr | % | Total | BBr | % | Total | BBr | % | Total | BBr | % | Total | BBr | % |
| Radius | 87 | 81 | 93 | 82 | 94 | 79 | 91 | 80 | 73 | 91 | 86 | 78 | 91 | 0 | 0 | n.a. | 74 | 67 | 91 | 0 | 0 | n.a. | 0 | 0 | n.a. | 87 | 79 | 91 |
| Ulna | 32 | 14 | 44 | 16 | 50 | 10 | 31 | 8 | 3 | 38 | 1 | 1 | 100 | 28 | 6 | 21 | 25 | 5 | 20 | 0 | 0 | n.a. | 0 | 0 | n.a. | 32 | 10 | 31 |
| Scaphoid | 14 | 12 | 86 | 10 | 71 | 10 | 71 | 1 | 1 | 100 | 11 | 9 | 82 | 2 | 0 | 0 | 1 | 1 | 100 | 3 | 1 | 33 | 5 | 5 | 100 | 6 | 4 | 67 |
| Lunate | 5 | 1 | 20 | 0 | 0 | 0 | 0 | 0 | 0 | n.a. | 4 | 0 | 0 | 5 | 0 | 0 | 0 | 0 | n.a. | 0 | 0 | n.a. | 0 | 0 | n.a. | 5 | 0 | 0 |
| Triquetrum | 14 | 4 | 29 | 2 | 14 | 1 | 7 | 2 | 1 | 50 | 11 | 1 | 9 | 13 | 1 | 8 | 6 | 1 | 17 | 1 | 0 | 0 | 2 | 0 | 0 | 11 | 1 | 9 |
| Pisiform | 0 | 0 | n.a. | 0 | n.a. | 0 | n.a. | 0 | 0 | n.a. | 0 | 0 | n.a. | 0 | 0 | n.a. | 0 | 0 | n.a. | 0 | 0 | n.a. | 0 | 0 | n.a. | 0 | 0 | n.a. |
| Trapezium | 5 | 0 | 0 | 3 | 60 | 0 | 0 | 3 | 0 | 0 | 4 | 0 | 0 | 0 | 0 | n.a. | 2 | 0 | 0 | 3 | 0 | 0 | 0 | 0 | n.a. | 2 | 0 | 0 |
| Trapezoid | 3 | 0 | 0 | 1 | 33 | 0 | 0 | 1 | 0 | 0 | 2 | 0 | 0 | 2 | 0 | 0 | 0 | 0 | n.a. | 1 | 0 | 0 | 0 | 0 | n.a. | 2 | 0 | 0 |
| Capitate | 6 | 3 | 50 | 3 | 50 | 3 | 50 | 1 | 1 | 100 | 4 | 2 | 50 | 4 | 1 | 25 | 4 | 1 | 25 | 1 | 1 | 100 | 1 | 1 | 100 | 4 | 1 | 25 |
| Hamate | 4 | 1 | 25 | 1 | 25 | 1 | 25 | 3 | 1 | 33 | 4 | 1 | 25 | 1 | 0 | 0 | 2 | 0 | 0 | 0 | 0 | n.a. | 0 | 0 | n.a. | 4 | 1 | 25 |
| Metacarpal I | 5 | 4 | 80 | 5 | 100 | 4 | 80 | 4 | 4 | 100 | 5 | 4 | 80 | 0 | 0 | n.a. | 2 | 2 | 100 | 5 | 4 | 80 | 0 | 0 | n.a. | 0 | 0 | n.a. |
| Metacarpal II | 2 | 1 | 50 | 1 | 50 | 1 | 50 | 0 | 0 | n.a. | 2 | 1 | 50 | 1 | 0 | 0 | 1 | 0 | 0 | 2 | 1 | 50 | 0 | 0 | n.a. | 0 | 0 | n.a. |
| Metacarpal III | 2 | 1 | 50 | 0 | 0 | 0 | 0 | 1 | 0 | 0 | 1 | 0 | 0 | 0 | 0 | n.a. | 2 | 0 | 0 | 2 | 0 | 0 | 0 | 0 | n.a. | 0 | 0 | n.a. |
| Metacrpal IV | 7 | 5 | 71 | 4 | 57 | 4 | 57 | 5 | 2 | 40 | 5 | 3 | 60 | 0 | 0 | n.a. | 5 | 2 | 40 | 5 | 3 | 60 | 1 | 0 | 0 | 1 | 1 | 100 |
| Metacarpal V | 9 | 6 | 67 | 6 | 67 | 5 | 56 | 3 | 3 | 100 | 8 | 4 | 50 | 1 | 0 | 0 | 6 | 4 | 67 | 8 | 4 | 50 | 0 | 0 | n.a. | 1 | 1 | 100 |
| Dig.I: prox. phalanx | 0 | 0 | n.a. | 0 | n.a. | 0 | n.a. | 0 | 0 | n.a. | 0 | 0 | n.a. | 0 | 0 | n.a. | 0 | 0 | n.a. | 0 | 0 | n.a. | 0 | 0 | n.a. | 0 | 0 | n.a. |
| Dig.I: dist. phalanx | 2 | 0 | 0 | 1 | 50 | 0 | 0 | 2 | 0 | 0 | 2 | 0 | 0 | 1 | 0 | 0 | 0 | 0 | n.a. | 2 | 0 | 0 | 0 | 0 | n.a. | 0 | 0 | n.a. |
| Dig.II: prox. phalanx | 1 | 0 | 0 | 0 | 0 | 0 | 0 | 0 | 0 | n.a. | 0 | 0 | n.a. | 0 | 0 | n.a. | 0 | 0 | n.a. | 1 | 0 | 0 | 0 | 0 | n.a. | 0 | 0 | n.a. |
| Dig.II: med. phalanx | 1 | 1 | 100 | 1 | 100 | 1 | 100 | 1 | 1 | 100 | 1 | 1 | 100 | 0 | 0 | n.a. | 1 | 1 | 100 | 1 | 1 | 100 | 0 | 0 | n.a. | 0 | 0 | n.a. |
| Dig.II: dist. phalanx | 1 | 1 | 100 | 0 | 0 | 0 | 0 | 1 | 0 | 0 | 0 | 0 | n.a. | 0 | 0 | n.a. | 0 | 0 | n.a. | 0 | 0 | n.a. | 0 | 0 | n.a. | 1 | 0 | 0 |
| Dig.III: prox. phalanx | 1 | 0 | 0 | 0 | 0 | 0 | 0 | 0 | 0 | n.a. | 0 | 0 | n.a. | 0 | 0 | n.a. | 0 | 0 | n.a. | 1 | 0 | 0 | 0 | 0 | n.a. | 0 | 0 | n.a. |
| Dig.III: med. phalanx | 2 | 1 | 50 | 2 | 100 | 1 | 50 | 2 | 1 | 50 | 2 | 1 | 50 | 0 | 0 | n.a. | 1 | 1 | 100 | 1 | 1 | 100 | 1 | 0 | 0 | 0 | 0 | n.a. |
| Dig.III: dist. phalanx | 1 | 0 | 0 | 0 | 0 | 0 | 0 | 1 | 0 | 0 | 0 | 0 | n.a. | 0 | 0 | n.a. | 0 | 0 | n.a. | 0 | 0 | n.a. | 0 | 0 | n.a. | 1 | 0 | 0 |
| Dig.IV: prox. phlanx | 1 | 1 | 100 | 1 | 100 | 1 | 100 | 1 | 1 | 100 | 1 | 1 | 100 | 0 | 0 | n.a. | 1 | 1 | 100 | 1 | 1 | 100 | 0 | 0 | n.a. | 0 | 0 | n.a. |
| Dig.IV: med. phalanx | 1 | 0 | 0 | 0 | 0 | 0 | 0 | 0 | 0 | n.a. | 1 | 0 | 0 | 1 | 0 | 0 | 1 | 0 | 0 | 1 | 0 | 0 | 0 | 0 | n.a. | 0 | 0 | n.a. |
| Dig.IV: dist. phalanx | 1 | 1 | 100 | 0 | 0 | 0 | 0 | 1 | 0 | 0 | 0 | 0 | n.a. | 0 | 0 | n.a. | 0 | 0 | n.a. | 0 | 0 | n.a. | 0 | 0 | n.a. | 1 | 0 | 0 |
| Dig.V: prox. phalanx | 1 | 1 | 100 | 0 | 0 | 0 | 0 | 0 | 0 | n.a. | 1 | 0 | 0 | 0 | 0 | n.a. | 0 | 0 | n.a. | 1 | 0 | 0 | 0 | 0 | n.a. | 0 | 0 | n.a. |
| Dig.V: med. phalanx | 3 | 1 | 33 | 1 | 33 | 1 | 33 | 1 | 1 | 100 | 3 | 1 | 33 | 2 | 0 | 0 | 2 | 1 | 50 | 3 | 1 | 33 | 0 | 0 | n.a. | 0 | 0 | n.a. |
| Dig.V: dist. phalanx | 1 | 1 | 100 | 0 | 0 | 0 | 0 | 1 | 0 | 0 | 0 | 0 | n.a. | 0 | 0 | n.a. | 0 | 0 | n.a. | 0 | 0 | n.a. | 0 | 0 | n.a. | 1 | 0 | 0 |
| All bones | **212** | **141** | 67 | **140** | 66 | **122** | 58 | **123** | **93** | 76 | **159** | **108** | 68 | **61** | **8** | 13 | **136** | **87** | 64 | **43** | **18** | 42 | **10** | **6** | 60 | **159** | **98** | 62 |

**Supplementary Table 1.** Evaluation of bone bruise (BBr) distribution in the hand and wrist fractures. Column two gives the number of fractures for each bone. Columns three to four provide the number and percentage of how often reader 1 (BBr_R1_) and reader 2 (BBr_R2_) rated BBr to be present in the case of fracture. Column five describes the number and percentage of fractures where both readers rated BBr to be present in a fracture (BBr_R1-R2_). Columns six to twelve provide an overview of the fracture features, including the number and percentage of BBr_R1-R2_ present in fractures with specific characteristics. The distribution demonstrated a higher frequency of BBr_R1-R2_ to be present in multifragmentary fractures (76%; column 6) compared to avulsion fractures (13%; column 8).
